# The shelf life of buffalo meat marinated with pomegranate (*Punica granatum*) peel extract

**DOI:** 10.5455/javar.2021.h552

**Published:** 2021-10-02

**Authors:** Nur Rasuli, Valentinus Priyo Bintoro, Agung Purnomoadi, Nurwantoro Nurwantoro

**Affiliations:** Department of Animal Science, Faculty of Animal and Agricultural Sciences, Diponegoro University, Semarang, Indonesia

**Keywords:** *Punica granatum*, buffalo meat, cold storage, antibacterial activity, peroxide value

## Abstract

**Objective:**

The purpose of this study was to investigate how pomegranate peel extract (PPE) can prevent lipid oxidation, peroxide value, and pathogenic bacteria growth in buffalo meat.

**Materials and Methods:**

PPE and buffalo meat were employed in this investigation. The buffalo meat marinated with PPE was evaluated by refrigerating it at a temperature of 5°C ± 1°C on days 0, 4, 8, 12, and 16. PPE was added to buffalo meat at a rate of 0% as a control (K0), 0.50% (K1), 1.00% (K2), 1.50% (K3), and 2.00% (K4).

**Results:**

The addition of PPE lowered the total plate count, peroxide value, lipid, and pH between treatments and storage period (*p* < 0.05). PPE’s high concentration of polyphenols, flavonoids, antioxidants, and antibacterial substances may decrease lipid oxidation, peroxide production, and bacterial growth rate.

**Conclusions:**

Marinating buffalo meat in PPE may help maintain the meat’s freshness while being stored at a refrigerator temperature (5°C ± 1°C).

## Introduction

Buffalo (*Bubalus bubalis*) is a meat-producing cattle commodity. Buffalo meat consumption has increased in recent years, in part due to its higher protein, fat, iron, and essential fatty acid content, as well as lower cholesterol [1,2] than other meats. Buffalo meat with high nutritional content is extremely sensitive to microbial infection, which can result in decomposition, foodborne illness, and shorter shelf life [3]. Bacterial activity during storage reduced the meat’s quality and oxidizing effect [4,5].

Lipid oxidation is the primary factor affecting the quality and shelf life of meat [6]. Efforts to inhibit microbial development and reduce lipid oxidation during storage are critical for the preservation of meat’s quality and safety.

Antioxidants are lipid oxidation inhibitors in meat and meat products, where they can delay the initiation of lipid oxidation by reacting with radicals and quenching metal ions. Preservatives for meat products have included synthetic antioxidants such as butylated hydroxylanisole, butylated hydroxyltoluene, and tertiary butylhydroquinone. The meat industry even uses chemical additives in several of its procedures to prevent dangerous bacteria from growing and to lengthen the shelf life of meat. Concerns concerning the safety of chemical additions have grown in recent years. Consumers are clamoring for the adoption of natural additives in place of food additives [7]. Therefore, it is worthwhile to investigate pomegranate peel extract (PPE) as a natural and safe alternative preservative.

Pomegranate peel is a natural component that is high in antioxidants [8,9] and antimicrobial [10]. Numerous studies have been conducted on the utilization of natural antioxidants to improve the oxidative stability of meat products [11,12]. Pomegranate peel antioxidants have been used to alleviate oxidative stress [13] and as an antibacterial agent [14–16]. Marination with natural components in food is a common strategy for reducing bacterial contamination with the primary purpose of extending the shelf life [17]. 

According to Schirmer et al. [18], marination can alter the physicochemical profile of meat, resulting in the eradication of bacteria that can cause decomposition to be delayed. As such, this study sought to determine the effect of pomegranate peel marination at various degrees on the total plate count (TPC), peroxide value, lipid, and pH of buffalo meat.

## Materials and Methods

### Pomegranate peel extract (PPE)

The peel of a ripe red pomegranate was employed in this investigation. The pomegranate peel was weighed and dried in a 50°C oven until it achieved a moisture content of 10%. It was then processed to create pomegranate peel powder. The following step was 48 h of marination in Aqua Dest at a ratio of 1:10 w/v. After that, the supernatant was separated from the residue using Whatman filter paper (no. 4). Following that, the supernatant was lyophilized (freeze-dried) until a single extract was obtained. This extract was dubbed PPE at the time [19].

### Polyphenol and flavonoid total test

The total polyphenol content of the extract was determined using the Folin–Ciocalteu technique. The polyphenol absorbance at 765 nm [20] and flavonoid absorbance at 430 nm [21] were determined using a spectrophotometer.

### Antioxidant activity of 2,2-Diphenyl-1-picrylhydrazyl (DPPH)

The DPPH scavenging activity was evaluated using the method published by Tangkanakul et al. [22], in which 0.15 ml of methanol extract from PPE was reacted with 0.1 mM DPPH solution (methanol solvent) up to 0.9 ml in a vial tube and vortex. The absorbance was then determined using a spectrophotometer set at 517 nm. The activity of DPPH free radical scavenging was presented in % scavenging activity (%SA) which was calculated as follows:

SA = 1–[(sample absorbance)/(absorbance control) × 100].

### Antibacterial activity test

The bacterial test consisted of three bacteria, namely *Escherichia coli* The American Type Culture Collection (ATCC) 25922*,* *Staphylococcus aureus* ATCC 25923, and *Salmonella enterica sv Enteritidis* ATCC 13076. The bacteria were incubated at 35°C for 2 h. The bacteria were then inserted into 5 ml of Brain Heart Infusion Broth and incubated for 24 h at 35°C. The antibacterial activity was measured following the method by Gullon et al. [23]. Bacteria suspension (about 50 μl) was put in a Petri dish, then filled with 20 ml of sterile agar nutrient and frozen. In a media which had been solidified, disc diffusion with a diameter of 3 mm was inserted. Each was filled with 20 μl sample solution (PPE) and was allowed to sit for 24 h at 37°C. Then, the inhibition zone diameter in the clear area of disc diffusion was measured using a caliper.

### Buffalo meat marination using PPE

The meat was obtained from Kudus District Slaughterhouse, Central Java Province, Indonesia. The slaughtered buffaloes were females of reproductive age (> 8 years old) weighing 300–350 kg. The first factor was 0% PPE marination (without PPE) and extract 0.5%, 1%, 1.5%, and 2% in a solution of 30% by weight of the sample (v/w). The second factor was the storage period (0, 4, 8, 12, and 16 days) in a refrigerator (± 5°C). The next step was the meat marination in PPE (based on the treatments). Each repetition used 100 gm of buffalo meat. After marination, the meat was drained for 10 min before being placed in a plastic bag and refrigerated (5°C). The parameter test was done on the sample during storage. The variables tested were total bacteria, rancidity test (peroxide), lipids, and pH. The total bacteria were calculated using the pour plate method following the Standard Nasional Indonesia method (SNI 2897) [24]; rancidity was determined by Shantha and Decker’s [25] peroxide value; lipid was determined by Soxhlet Method Lipid Level Analysis (Association of Agricultural Chemists) [26], and meat pH was determined by the method of Ockerman [27].

### Statistical methods

The experiment was set up as a 5 × 5 factorial design, with the following factors: i) five PPE concentrations: 0%, 0.5%, 1.0%, 1.5%, and 2.0%; and ii) five storage periods: 0, 4, 8, 12, and 16 days in the refrigerator (5°C). The experiment was repeated twice, with three repetitions, and all parameters measured had two duplications. Data were analyzed using the general linear model procedure of IBM SPSS Statistics 21 (IBM SPSS, Chicago, IL). Antibacterial activity was calculated on PPE concentration levels, and three types of pathogen bacteria, while the marinated effect on buffalo’s meat was TPC, peroxide value, lipid, and pH. The data were statistically analyzed using the Duncan-Test at 5%.

## Results and Discussions

### Yield, total polyphenol, flavonoid, and antioxidant

The extract produced a yellow color and a certain plant scent. The yield value was 30% (Table 1), which is in accordance with Kanoun et al. [28] showing that the water extract was higher than the methanol extract (46.08%, 0.155%, 34.92%, and 0.33%). This was shown by a high level of solubility and water polarity. Water becomes the best solution to extract a chemical substance from a different fruit peel.

**Table 1.** Yield, phytochemicals, and antioxidant content of PPE.

Parameter	PPE
Yield (%)	30.0
Flavonoid (mg/gm)	35.0
Polifenol (mg/gm)	217.8
Antioksidan IC_50_ (ppm)	9.1

The polyphenol compound is the most important substance in fruit, which indicates some functional characteristics such as antibacterial and antioxidant capacity. The results also revealed that PPE contains 217.8 mg/gm of total polyphenol and 35 mg/gm of total flavonoid. This polyphenol content was different from that previously reported in [29–32]. Each researcher reported 165.4, 161.3, 158.5, and 209.8 mg/gm of the equivalent of gallic acid, PPE, with a water solution. Flavonoid content was also different from what Abid et al. [32] reported, ranging from 9.98 to 15.25 mg/gm of Quercetin equivalent.

The result showed PPE contained antioxidant IC_50_ 9.1 ppm, so the PPE is considered a very strong antioxidant due to IC_50 _value [33]. The pomegranate peel has higher antioxidant activity than the rest of the fruit [34]. 

### Antibacterial activity

The antibacterial activity of PPE at concentrations of 0.5%, 1.0%, 1.5%, and 2.0% is presented in Table 2, which shows significantly different results (*p* < 0.05). Treatment with 2.0% concentration had the highest antibacterial activity, which was marked by a high diameter of inhibition zone, followed by treatments of 1.5% concentration and 1.0%. The lowest antibacterial activity was the treatment with 0.5% concentration. The diameter of the inhibition zone, which ranged from 7 to 10 mm in *E. coli* ATCC 25922, 6 to 11 mm in *S. aureus* ATCC 25923, and 8 to 11 mm in *S. enterica *sv* Enteritidis* ATCC 13076, was used to calculate the value of antibacterial activity. Pomegranate peel powder’s high polyphenol content can inhibit the bacteria *E. coli*, *S. aureus*, *Listeria monocytogenes*, and *Salmonella* sp. [23]. In a different research, Al-Zoreky [35] stated that the use of PPE with water extract did not show any antibacterial activity in *E. coli *and *S. aureus*. The result of this study showed that the extract of pomegranate showed antibacterial activity in three strains that were tested. The activities observed were different according to the concentration of extract used. This result was agreed with a previous study that reported that the difference in extract effect was a result of different extraction methods [36].

**Table 2.** Antibacterial activity of PPE.

Concentration	*Escherichia coli* ATCC 25922	*Staphylococcus aureus* ATCC 25923	*Salmonella enterica sv Enteritidis* ATCC 13076
(%)	Inhibition zone diameter (mm)		
0.5	7.0 ± 1.0^c^	6.0 ± 1.0^b^	8.0 ± 1.0^b^
1.0	8.0 ± 1.0^bc^	7.0 ± 1.0^b^	8.0 ± 1.0^b^
1.5	9.0 ± 1.0^ab^	10.0 ± 1.0^a^	9.0 ± 1.0^b^
2.0	10 ± 0.0^a^	11.0 ± 1.0^a^	11 ± 1.0^a^

Different superscripts between columns (a, b, c,) showed significant differences (*p* < 0.05).

### Microbiological characteristics of buffalo’s meat 

Table 3 shows the average quantity of microorganisms found in buffalo meat after marinating in PPE. The meat marinated in 0.5%, 1.0%, 1.5%, and 2.0% PPE showed that the storage length and concentration are significantly different (*p* < 0.05) depending on the total bacteria in buffalo meat. The use of PPE in buffalo meat has been shown to retain the meat’s quality for up to an 8-day storage period. PPE’s antibacterial activity, which may inhibit both Gram-positive and Gram-negative bacteria due to antimicrobial compounds found in pomegranates such as organic acid and polyphenol, most likely delayed bacteria’s growth in the marinated meat [37,38]. Additionally, pomegranates contain polyphenols such as punicalagin and punicalin, which have a strong antimicrobial effect [39]. According to Endo et al. [40], the tannin or polyphenol content of PPE may serve as a protein depositor, inhibiting bacterial development. Additionally, the death of the microbial cell was induced by the selective inhibition of microbe ATP synthase as a result of polyphenol activity, which resulted in the loss of cellular energy in germs [41]. The duration of storage, the temperature, and the packaging all have an effect on bacterial populations [42]. Additionally, because PPE contains ellagic acid, it qualifies as an acidification that inhibits bacterial development.

**Table 3.** Effect of PPE on TPC, peroxides value, lipid and pH of buffalo meat at different storage periods.

Treatment	Storage time (days)				
	0	4	8	12	16
TPC (colony-forming unit/gm)					
K0	4.1 × 10^5 h^	3.4 × 10^6 fgh^	7.4 × 10^6 cde^	1.1 × 10^7 ab^	1.1 × 10^7 a^
K1	4.0 × 10^5 h^	4.6 × 10^5 h^	3.4 × 10^6 fgh^	8.3 × 10^6 abcd^	1.0 × 10^7 abc^
K2	1.6 × 10^5 h^	2.0 × 10^5 h^	1.0 × 10^5 h^	5.5 × 10^6 def^	7.4 × 10^6 cde^
K3	3.4 × 10^5 h^	1.8 × 10^5 h^	5.5 × 10^5 h^	9.4 × 10^6 abc^	4.4 × 10^6 efg^
K4	2.3 × 10^5 h^	4.8 × 10^5 h^	5.5 × 10^5 h^	8.2 × 10^6 bcd^	2.0 × 10^6 gh^
PV (mEqO_2_/kg)					
K0	0.116^h^	2.858^ef^	5.345^cd^	6.572^ab^	6.978^a^
K1	0.104^h^	0.233^gh^	1.362^fgh^	5.237^bcd^	5.334^bc^
K2	0.099^h^	0.142^h^	1.706^fg^	3.771^de^	4.203^cde^
K3	0.120^h^	0.217^gh^	1.718^fg^	4.037^cde^	3.845^cde^
K4	0.090^h^	0.206^gh^	1.360^fgh^	3.328^e^	2.764^ef^
Lipid (%)					
K0	0.94^a^	0.78^abcdef^	0.72^cdefg^	0.68^efg^	0.59^g^
K1	0.73^bcdefg^	0.76^bcdef^	0.64^fg^	0.82^abcde^	0.86^abcd^
K2	0.74^bcdefg^	0.62^fg^	0.85^abcd^	0.78^abcdef^	0.89^ab^
K3	0.79^abcdef^	0.72^cdefg^	0.76^bcdef^	0.75^bcdefg^	0.88^abc^
K4	0.70^defg^	0.70^defg^	0.71^defg^	0.74^bcdefg^	0.84^abcde^
pH					
K0	6.41^efg^	6.55^cd^	6.65^bc^	6.75^ab^	6.80^a^
K1	6.36^efgh^	6.24^hi^	6.41^efg^	6.42^defg^	6.48^ed^
K2	6.36^efgh^	6.21^i^	6.46^def^	6.33^fghi^	6.35^efgh^
K3	6.41^efg^	6.25^hi^	6.62^c^	6.31^ghi^	6.39^efg^
K4	6.31^efg^	6.36^efgh^	6.37^efgh^	6.31^ghi^	6.34^fghi^

^a,b,c,d,e,f,g,h,i^ Superscripts in the column show significant (*p* < 0.05).

K0: 0.0% *Punica granatum* extract concentration (control), K1: 0.5% *P. granatum* extract concentration, K2: 1.0% *P. granatum* extract concentration, K4: 1.5% *P. granatum* extract concentration, K4: 2.0% P. granatum extract concentration.

D0: day 0, D4: day 4, D8: day 8, D12: day 12, D16: day 16.

### Peroxide value

The peroxide value (Table 3) demonstrated no difference in PPE between the K0, K1, K2, K3, and K4 treatments with a storage time of 0 days (*p* > 0.05), but a difference (*p* < 0.05) between the combinations of K0D4 and KOD8 with K1D4, K2D4, K3D4, and K4D4 and K1D8, K2D8, and K4D8. The control samples had the greatest peroxide value after 4 days of storage, at 2.858 mEqO_2_/kg. Meanwhile, samples of buffalo meat with K4 PPE 0.206 mEqO_2_/kg had the lowest peroxide value. The maximum level of peroxide was detected in the control sample after 16 days of storage, at 6.978 mEqO_2_/kg. The peroxide value of meat-added PPE increased significantly after 12 days of storage, reaching 5.237 mEqO_2_/kg in K1 PPE. The greater the PPE concentration in buffalo meat, the lower the peroxide concentration. According to Drinić et al. [8], PPE had an effect on the peroxide value.

After the 16th day, the peroxide value of 1.5% and 2% extracts declined. It was determined that the hydroperoxide degradation occurred via breakdown of the hydroperoxide to create secondary lipid oxidation products [31]. Peroxide decomposes into a stable product such as aldehydes (malondialdehyde, hydroxynonenal, and hydroxyhexanal), ketone, hydrocarbon, epoxy, alcohol, and other organic compounds as a secondary product of lipid oxidation [43]. Turgut et al. [5] reported that PPE might inhibit lipid oxidation and prevent the generation of lipid peroxide and malondialdehyde. The maximum amount of peroxide that can be tolerated in a food product is between 10 and 20 mEqO_2_/kg [44].

### Lipid level

There was an increase in the lipid content of buffalo meat marinated with PPE at concentrations of 0.0%, 0.5%, 1.0%, 1.5%, and 2.0% for 0, 4, 8, 12, and 16 days, respectively. Meanwhile, lipid levels tend to decrease in control samples. Lipid oxidation may occur as a result of reduced lipid levels in storage. Meanwhile, an increase in lipid concentration could be the result of a change in proportion produced by the PPE addition and storage duration. The longer the storage period, the lower the protein concentration. Additionally, the active ingredient content of pomegranate extract in the form of antioxidants may limit the oxidation of lipids in buffalo meat, as evidenced by the quantity of stable lipid level. The active ingredient content in the form of pomegranate extract inhibited the oxidation of buffalo meat lipids, as demonstrated by the relatively stable lipid level. PPE’s antioxidant action has been linked to its phenolic composition [31]. A phenolic antioxidant inhibits the production of free radicals that are devoid of lipids. This radical participates in the oxygen reaction or absorption during the process of autoxidation [45]. The phenolic compound, according to Kanatt et al. [29], has the ability to bind metal ions. Thus, by attaching to free radicals, notably iron and copper, phenolates can prevent their generation and spread [45].

### pH value

The data in Table 3 indicated that the PPE notation (a) had no effect on the extract levels K0, K1, K2, K3, and K4 throughout a 0-day storage period (*p* > 0.05). However, there was a difference in the 4-day storage time between K0D4 and K1D4, K2D4, K3D4, and K4D4 (*p* < 0.05). The increase in the meat’s pH value suggested an increase in the bacteria population [46]. In comparison, the pH of buffalo meat treated with PPE was lower than the control. This showed that the rate of microbial decomposition of their meat had decreased [47], which was verified by prior study on avian meat [48]. This occurred as a result of pomegranate’s antibacterial properties, which include organic acid and polyphenol [38].

According to the findings of this study, buffalo meat on average had a higher pH, ranging from 6.21 to 6.62, than buffalo meat on a normal basis, which has a pH of 5.63 to 5.76. This disparity could be explained by the water buffalo’s employment as a working animal. According to Rao et al. [49], the high value of buffalo meat is due to the animal’s use as a working animal, which contributed to the pH level fall. Additionally, a high pH value could have been generated by an age greater than 8 years. According to Mendrofa et al. [50], elder buffalo have a higher meat pH than younger buffalo. According to Weglarz [51], the change in meat pH is primarily influenced by intrinsic factors such as muscle type, species, and individual condition of the livestock, as well as extrinsic factors such as handling of livestock prior to and following slaughter, environmental temperature, additive substances, and stress. According to Naveena et al. [52], the pH of buffalo meat is approximately 5.56, whereas the pH of cow flesh is approximately 5.47. According to Aberle et al. [53], livestock that is not rested generate meat with a dark hue, a hard texture, dry flesh, and a high pH value. Ilavarasan et al. [54] discovered a correlation between the pH value and color of meat. The higher pH value resulted in a darker color and a drier surface of the meat, indicating that the flesh retains its moisture more effectively.

## Conclusion

Marination of buffalo’s meat using PPE solution for 15 min can maintain the quality of buffalo’s meat for 0 to 8 days storage at refrigerator temperature ± 5°C. Two percent concentration of PPE gives the best result because this concentration can suppress the total content of bacteria as required by SNI 3932.

## List of abbreviations

ATCC, The American Type Culture Collection; °C, Degrees celsius; PPE, Pomegranate peel extract; TPC, Total plate count; ±, Plus-minus sign; ATP, Adenosine triphosphate; SA, Scavenging activity; sv, Serovar or serotipe. 
